# Improved Ablation Resistance of Silicone Rubber Composites by Introducing Montmorillonite and Silicon Carbide Whisker

**DOI:** 10.3390/ma9090723

**Published:** 2016-08-24

**Authors:** Guangwu Zhang, Fuzhong Wang, Zhixiong Huang, Jing Dai, Minxian Shi

**Affiliations:** 1 Key Laboratory of Advanced Technology for Specially Functional Materials of Ministry of Education, School of Materials Science and Engineering, Wuhan University of Technology, Wuhan 430070, China; gwzhang@whut.edu.cn (G.Z.); fzwang@whut.edu.cn (F.W.); daijing@whut.edu.cn (J.D.); 2 School of Materials Science and Engineering, Qilu University of Technology, Jinan 250353, China

**Keywords:** silicone rubber, ablation resistance, mechanical properties, montmorillonite, silicon carbide whisker

## Abstract

Montmorillonite (MMT) was added to silicone rubber (SR) to improve the ablation resistance of the silicone. Following this, different quantities of silicon carbide whiskers (SiCw) were incorporated into the MMT/SR to yield a hybrid, ablative composite. The tensile strength and elongation at break of the composite increased after the addition of MMT. The ablation test results showed that MMT helped to form a covering layer by bonding with the silica and other components on the ablated surface. The linear and mass ablation rates exhibited decreases of 22.5% and 18.2%, respectively, in comparison to a control sample. After further incorporation of SiCw as the second filler, the resulting composites exhibited significantly higher tensile strength and ablation resistance, but not particularly lower elongation at break in comparison to the control sample. The SiCw/MMT fillers were beneficial in forming a dense and compact covering layer that delayed the heat and oxygen diffusion into the inner layers, which improved the ablation properties effectively. The remaining whiskers acted as a micro skeleton to maintain the composite’s char strength. Compared to the control sample, the linear and mass ablation rates of the composite after incorporating 6 phr SiCw and 10 phr MMT decreased by 59.2% and 43.6%, respectively. These experimental results showed that the fabricated composites exhibited outstanding mechanical properties and excellent ablation resistance.

## 1. Introduction 

Polymeric ablative materials play a strategic role in the thermal protection system of rocket nozzles, space vehicles and rocket combustion chambers [1,2,3]. Ablation is the sacrifice of the material to form a stable surface char that protects the internal part of materials from high temperature flame [4]. Therefore, ablative materials should have good ablation resistance to endure severe high-temperature conditions. Thermosetting resins such as phenolic-based composites are widely used as ablative materials because of the high char yield generated by the resin matrix [5,6,7]. However, ablatives should also be flexible in some applications to resist the high stress produced by combustion gases. As a consequence, elastomeric ablative materials are generally used as heat insulators in combustion chambers [8,9,10,11]. Usually, rubbers such as ethylene-propylene-diene terpolymer (EPDM) and nitrile butadiene rubber (NBR) are favored matrices as insulation materials because of their appropriate mechanical strength, excellent thermal stability, and easy processing [12,13].

Rubber alone cannot form a strong char layer and survive the severe high-temperature environment. Therefore, inorganic fillers (e.g., silica, metal oxides, layered clay, or carbonaceous fillers) are incorporated into the elastomer matrices to improve the ablation performance [14,15]. The incorporation of clay filler not only improves mechanical properties but also other properties such as wear abrasion, hydrophobicity, and flammability of the rubber-based composites [16,17,18,19,20,21]. In addition, clay has also been proven to be effective in improving the ablation resistance of elastomer-based composites. For example, Iqbal et al. [22] used modified nanokaolinite to impregnate acrylonitrile butadiene rubber, resulting in a significant enhancement in the ablation resistance and reduction in the temperature elevation at the back face of the nanocomposite. Guan et al. [14] reported a decrease in the linear ablation rate and an increase in the mass ablation rate of a montmorillonite (MMT)-reinforced hydrogenated nitrile butadiene rubber (HBNR) composite compared to silica or expanded graphite reinforced composites. He et al. [23] found that the dispersion of MMT in the elastomer matrix played an important role in the ablation performance of MMT-filled EPDM composites. 

Silicone rubber (SR) is one of important organic silicon products. Silicone rubbers are linear polysiloxanes, which can be classified according to the curing process as high-temperature vulcanized (HTV) silicone rubber and room-temperature vulcanized (RTV) silicone rubber, respectively [24,25]. HTV silicone rubbers are high viscosity solid gums (molar mass about 500,000 g/mol) with less than 0.5 mol % of vinylsiloxane units, usually cured with peroxides such as 2,5-bis(tert-butyl peroxy)-2,5-dimethyl hexane (DBPMH) [26]. Curing takes place between the Si-CH = CH_2_ groups in a free radical crosslinking reaction in the presence of peroxide. RTV silicone rubbers are available in the type of liquid, and the crosslinking mechanism is commonly thought of as hydrosilation and a condensation reaction. Addition-type liquid (ATL) silicone rubbers are mainly provided as two components called part A and part B. The vinyl-containing silicones are reacted through hydrosilation with hydrosilane materials as crosslinking agents and have platinum compounds as a catalyst [27,28]. The latter of RTV silicone rubbers falls into two classes, namely one-part and two-part systems. In the one-part system, silicone chain contains a plurality of hydrolysable groups such as acetoxy or oxime groups, which readily react with atmosphere moisture to form silanol groups that allow further condensation [24,25]. In the two-part system, silicone chain is end-capped with hydroxyl groups, usually using tetraethoxysilane as crosslinking agent to form three-dimensional network structure [24,25].

Because SR has high thermal stability and flexibility together with chemical resistance [24,29], SR can be regarded as a good insulation material candidate. There are many reports of SR-based ablative composites. Dow Corning Corporation developed a commercial room-temperature vulcanized (RTV) silicone rubber (RTV-SR) composite DC 93-104, as an ablative coating [30]. Zou et al. [31,32] demonstrated an improvement in the ablation resistance of aluminum silicate ceramic fiber/high-temperature vulcanized (HTV) silicone rubber (HTV-SR) composites by adding wollastonite or calcium carbonate. Recently, some ultra-high melting ceramics, for example, zirconia (ZrO_2_) and zirconium carbide (ZrC), were incorporated into HTV-SR composites to evaluate the resulting ablation properties [15]. Although the incorporation of inorganic fillers can make the char layer more compact, SR ablative composites also have some disadvantages. Usually, high quantities of dense inorganic fillers are added to elastomers to develop the desired properties in the resulting composite. This can lead to a significant decrease in flexibility and a considerable increase in density. Therefore, a single type of inorganic filler is not sufficient to meet the ablation and mechanical property requirements of SR insulators. 

Silicon carbide whiskers (SiCw) have generally been used as a reinforcing material in ceramic, metal and polymer matrices because of their outstanding oxidative stability, high temperature strength and high thermal shock resistance [33,34,35]. Moreover, SiCw can keep their whisker shape even after they are fully or partial oxidized. In addition, SiCw have a good wettability for silica because of the coated silica layer derived from the oxidation of SiCw at high temperature [34,35]. This is beneficial for increasing compactness of the char layer. However, there are limited reports in the literature of the ablation performance of polymer composites containing SiCw. Consequently, in this reported study, SiCw was used as a second-phase filler to improve the ablation resistance of SR composites.

In this reported work, we chose high-viscosity solid silicone gums as elastomer matrix, using common peroxide (specifically, DBPMH) as curing agent. MMT was incorporated into SR to improve the ablation resistance and then SiCw was included as the second-phase filler and for the preparation of SiCw/MMT hybrid SR composites. The incorporation of small quantities of SiCw was expected to further improve the ablation resistance of the SR composites and produce no significant decrease in flexibility and increase in density at the same time. The effects of MMT and SiCw on the mechanical and ablation properties of SR composites were investigated in detail.

## 2. Experimental

### 2.1. Materials

**Figure 1:**
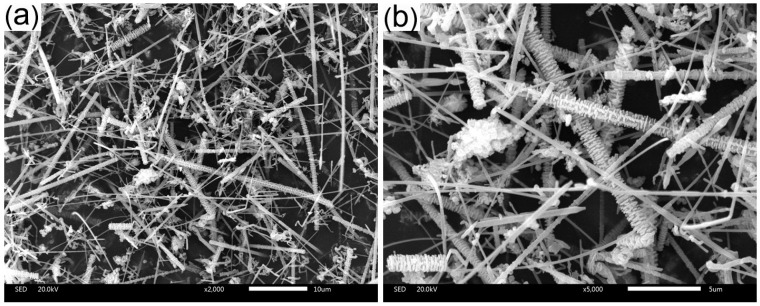
Scanning electron microscopy (SEM) images of SiC whiskers (SiCw): (**a**) low magnification; (**b**) high magnification.

Methyl vinyl silicone rubber (MVS, 110-2; Mn = 500,000–700,000; vinyl content = 0.15–0.18 mol %) was provided by BlueStar Chengrand Research Institute of Chemical Industry (Chengdu, China), methyl phenyl vinyl silicone rubber (MPVS, ASIBO-129-1; Mn = 500,000; the ratio of phenyl to methyl = 20%; vinyl content = 0.16 mol %) was purchased from Shanghai Asibo Silicone Co., Ltd. (Shanghai, China). And 2,5-bis(tert-butyl peroxy)-2,5-dimethyl hexane (DBPMH, Aladdin Industrial Inc., Shanghai, China) was used as the curing agent. Fumed SiO_2_ (R106, Degussa, Essen, Germany) was used to improve the mechanical performance of the composites. Montmorillonite (MMT, Charex.44PSS) was kindly supplied by Nanocor Inc. (Hoffman Estates, IL, USA). Silicon carbide whiskers (SiCw; SiC content >96%, volume of whisker >90%) was obtained from Changsha Sinet Advanced Materials Co., Ltd. (Changsha, China). The diameter of the whiskers was 0.1~1 μm, and the length was 10~50 μm. The morphology of SiCw is shown in Figure 1. Short phenolic fiber (Kynol) was produced by Gun EI Chemical Industry Co., Ltd. (Gunma, Japan). Tetrahydrofuran (THF) was purchased from Sinopharm Chemical Reagent Co., Ltd. (Shanghai, China).

### 2.2. Preparation of MMT/SR Masterbatch

MVS and MPVS (mass ratio 4:6) were first completely dissolved in THF, followed by the addition of MMT. The mass ratio of MMT to total SR was 25:75. Subsequently, the mixture was processed by mechanical mixing at a speed of 1300 rpm for 3 h. Then, the THF was evaporated off at 65 °C with stirring and the mixture was dried in vacuum at 60 °C for 24 h.

### 2.3. Fabrication of SR Composites

The compounds were processed in a two-roll open mixing mill at room temperature. The SR and MMT/SR masterbatch were added first and then relevant additives were added progressively. Kynol fibers were slowly added to the compound to reduce the breakdown of the fibers. Then DBPMH was added as the last component. The detailed formula is listed in Table 1. The SR compounds were compression-molded at 165 °C for 15 min under 10 MPa. The ablators were vulcanized in a mold with diameter of 30 mm and thickness of 10 mm. The secondary vulcanization was carried out at 180 °C for 2 h in an airflow drier.

### 2.4. Characterization

#### 2.4.1. Ablation Test

**Figure 2:**
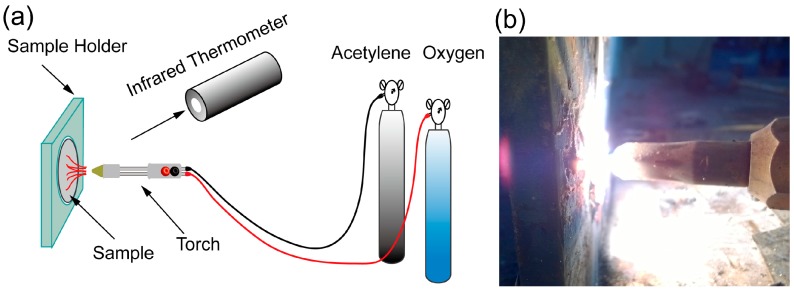
(**a**) Schematic of the ablation test and (**b**) specimen under the test.

The ablation performance of the SR composites was evaluated by oxyacetylene torch test. The vertical distance of the nozzle to the sample was 10 mm and the inner diameter of the nozzle was 2.0 mm. The surface temperature of the samples was approximately 2200 °C and was monitored using an infrared thermometer. The testing time was fixed at 20 s. Figure 2 shows a schematic diagram of the ablation test and specimen under the test. The linear and mass ablation rates were calculated according to the following formulae. 

Linear ablation rate,

(1) $$R_{l}=\left(d_{1}-d_{2}\right)∕t$$

Mass ablation rate,

(2) $$R_{m}=\left(m_{1}-m_{2}\right)∕t$$

where *d*_1_, *m*_1_, and *d*_2_, *m*_2_ are the thickness and mass of the sample before and after ablation test, respectively, and *t* is the testing time. The *d*_2_ thickness was measured after removing the char layer.

#### 2.4.2. X-ray Diffraction (XRD)

The X-ray diffraction (XRD) patterns of MMT powder, SR composites, and the char powders were obtained using by a Bruker AXS D8 Advance diffractometer (Karlsruhe, Germany) with Cu Kα radiation at room temperature. 

#### 2.4.3. Morphology

The ablated surfaces of the SR composites were examined using a scanning electron microscopy (SEM) JSM-5610LV (JEOL Ltd., Tokyo, Japan). And the cross section of the char layers was characterized by field emission scanning electron microscopy (FESEM, Zeiss Ultra Plus, Carl Zeiss NTS GmbH, Oberkochen, Germany). The surface and cross section of char samples were coated with a thin layer of gold prior to imaging.

#### 2.4.4. Mechanical and Physical Properties

The tensile strength and elongation at break of the composites were measured on Instron-4465 (Instron Engineering Co., Norwood, MA, USA) universal testing machine according to GB/T 528-2009 [36] at room temperature. The crosshead speed was 500 mm/min. The dimension of the dumbbell specimens was 75 mm × 4 mm × 1.6 mm. For each group of composites, a minimum of three specimens were tested.

The Shore A hardness of the composites was measured using a LX-A BY-4040A apparatus (Jiangdu Boyu Testing Machine Factory, Yangzhou, China) according to GB/T 531.1-2008 [37] at room temperature. The samples were 6 mm thick and the test results that are listed below were averages of a minimum of five tests.

The density of the composites was measured according to GB/T 533-2008 [38] at 23 °C. The reported results were the average of three specimens.

#### 2.4.5. Thermogravimetric Analysis (TGA)

Thermogravimetric analysis was carried out using a TGA STA449c/3/G (NETZSCH Group, Selb, Germany) in an air environment at a heating rate of 10 °C/min from room temperature to 800 °C.

## 3. Results and Discussion

**Figure 3:**
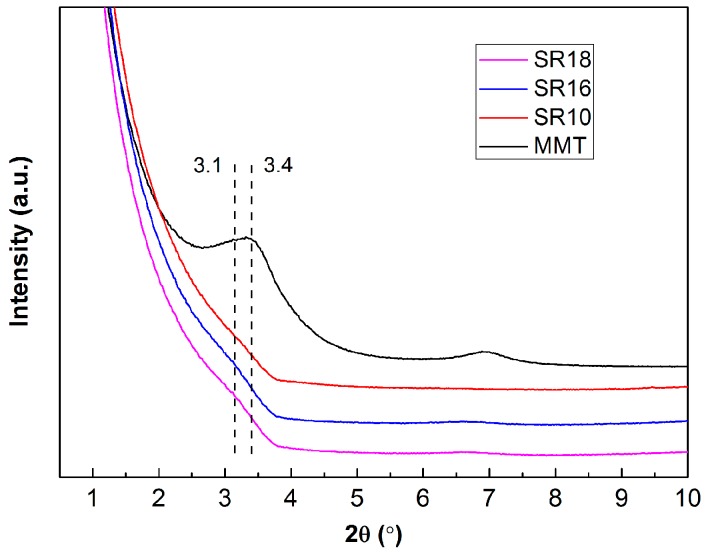
X-ray diffraction (XRD) patterns for Charex.44PSS MMT and the composites containing MMT or SiCw/MMT fillers.

Figure 3 shows the XRD patterns of MMT and the various composites containing the MMT filler. In the MMT spectrum, the diffraction peak at 2θ = 3.4° corresponds to the interlayer d-spacing (d_001_) of the organic modified MMT (25.0 Å). In this work, the solution blending method was used to prepare the masterbatch at a high mechanical mixing speed with sufficient time so that the intercalated/exfoliated MMT structures in the SR matrix were obtained [21]. The different samples containing MMT did not exhibit a crystalline peak, suggesting that no significant stacking of platelets occurred in the composites [10,39]. However, the composites SR16 and SR18 exhibited some increase of intensity in the low angle of about 2θ = 3.1°. It is clear that the interlayer spacing between the MMT layers increased after being introduced into the SR matrix, meaning that the silicone chains had penetrated into the MMT interlayers [40]. The XRD analysis provides a sense of the state of the MMT in the SR matrix, but it is not sufficient to validate the exfoliation of the MMT layers. In other words, this blending method is an effective way to obtain an intercalation of MMT layers and perhaps a degree of exfoliation.

**Figure 4:**
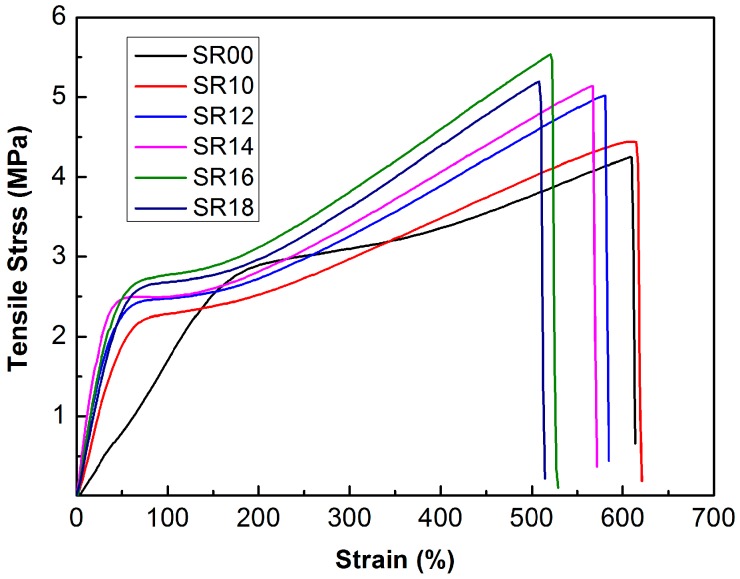
Representative stress-strain curves of SR composites.

It is well known that a combustion chamber can suffer severe thermal deformation under the high shear stress caused by the burning of a propellant. Therefore, a high tensile strength and high elongation at break in the elastomeric ablative materials is needed to accommodate this eventuality. The resulting mechanical properties of the SR composites are shown in Figure 4 and Table 2. Introducing MMT into the SR system slightly increased the tensile strength and elongation at break. The tensile strength of SiCw/MMT-filled composites increased progressively with the increasing amount of SiCw, reaching a maximum value (5.61 MPa) at 6 phr SiCw, but the elongation at break decreased. The SiCw/MMT particles appeared to physically entangle with rubber chains, which restricted the mobility of the chain, resulting in an increase in tensile strength, but a decrease in flexibility. Meanwhile, the particles tended to form agglomerates with increasing SiCw quantities. These agglomerates acted as points of high stress concentration which were prone to breakage, resulting in local defects and facile mechanical failure of the composite [41]. Fortunately, even after adding 8 phr SiCw into the 10 phr MMT-reinforced SR system, the elongation at break of this composite remained above 500% and the tensile strength was higher than 5 MPa. The elongation at break is much higher than the results reported by Yang et al. [15], where this value of their SR composite was below 100% after the incorporation of 40 phr ZrC or ZrO_2_. Hence, the mechanical properties of the SR composites fabricated in this reported effort are quite sufficient to meet the requirements of elastomeric ablative materials.

The density of the composites is also a key criterion for evaluating the quality of elastomeric ablative materials. On the basis of ablation performance, the density of the composite should be as low as possible, meaning that low quantities of a low density filler would be preferable. The densities of MMT and SiCw are 1.7 g·cm^−3^ and 3.2 g·cm^−3^, which are relatively low when compared to ZrC (6.7 g·cm^−3^), ZrO_2_ (5.9 g·cm^−3^), ZrB_2_ (6.08 g·cm^−3^) and TiB_2_ (4.52 g·cm^−3^). Introducing even a small amount of MMT and SiCw into the rubber can improve the resulting ablation properties significantly, while the density of the composites will increase marginally with increasing filler contents. The density of all the fabricated composites was less than 1.20 g·cm^−3^. The low density of the elastomeric insulator is very beneficial in aerospace applications, particularly for thrust control and the upper stages of rockets. 

The Shore A hardness of the composites is displayed in Table 2. The hardness value of the SR10 composite was increased slightly to 1.7 compared to the control sample. When employing SiCw as the second filler, the Shore A hardness of the composites increased considerably with increasing SiCw content, due to the very rigid and stiff nature of the SiCw inorganic filler. 

**Figure 5:**
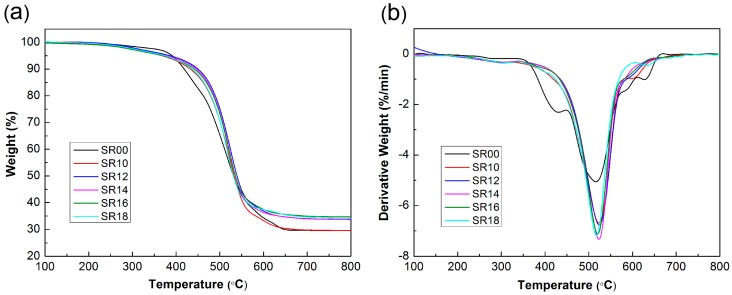
Thermogravimetric analysis (TGA) (**a**) and differential thermogravimetric analysis (DTGA) (**b**) curves of SR composites measured in air atmosphere.

The thermal oxidation stability of SR composites is shown in Figure 5 and Table 3. The initial decomposition temperature (T_0.1_, temperature at 10% weight loss), the temperature of the maximum decomposition rate (T_max_) and the residual weight (R_800_) of the samples are listed in Table 3. As can be seen in Figure 5, the weight loss of SR00 took place in three steps in air atmosphere. In the range of 350~450 °C, a considerable weight loss can be seen due to the sidechain oxidation of MVS and MPVS. A significant weight loss occurred in the range of 450~550 °C where the siloxane chains and phenolic main chains were broken and a large quantity of gaseous product was generated. Above 550 °C, the weight loss was attributed to the oxidation of the polymeric char resulting from the decomposition of the matrix and phenolic at high temperature. After adding MMT to the composite, a small amount of weight loss below 350 °C was observed due to the oxidation and decomposition of the organic modifier of MMT. In the intermediate range of 350~550 °C, the composite containing MMT showed a significant delay in weight loss that may have resulted from the physical barrier provided by the MMT, which could retard both the escape of decomposition gases and the diffusion of oxygen into the interior of the material [42]. Above 550 °C, the rearrangement of the silicate layers combined with the oxidation of the polymeric char that resulted from the decomposition of the matrix and phenolic were responsible for the subsequent weight loss. After adding SiCw to the composites, the TGA and DTGA curves of the resulting composites showed no obvious differences with the curves for the SR10 composite below 550 °C. Then, as the temperature was elevated, it appeared that the addition of SiCw improved the stability of the composites. However, the residual weight of the composites did not show any obvious change with increasing SiCw content.

**Figure 6:**
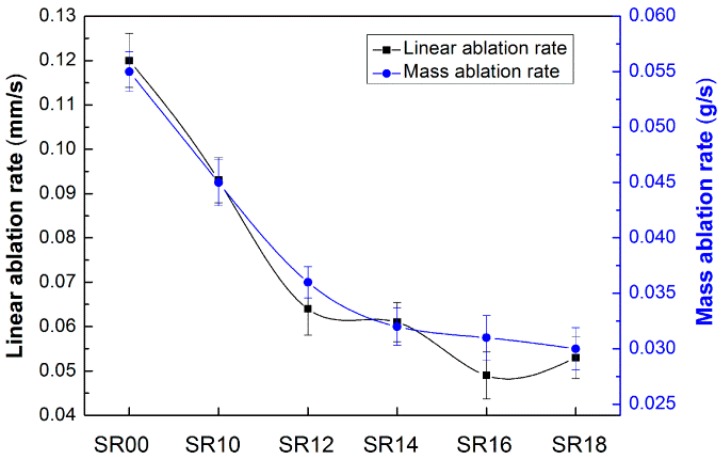
Characteristic ablation results of SR composites.

Generally, the ablation-resistant abilities of the composites could be characterized with linear and mass ablation rates after an oxyacetylene torch test. Figure 6 depicts the characteristic ablation results of the SR composites. The incorporation of the MMT filler alone resulted in a limited enhancement of the ablation performance of the composite, specifically, decreases of 22.5% and 18.2% in linear and mass ablation rates over the control sample, respectively. The ablation properties of the composites were greatly increased after the addition of SiCw. When the SiCw content was 6 phr, the linear and mass ablation rates attained values of 0.049 mm/s and 0.031 g/s, which were reduced by 59.2% and 43.6% compared to the control sample, respectively. Afterwards, the composite exhibited a decrease in mass ablation rate, but an increase in linear ablation rate. It is well known that carbides can be oxidized to generate an oxide and a byproduct. The oxide layer can prevent further oxidation of the carbide. The oxidized SiCw can improve the tenacity of a char layer on the microscale and comingle with the other components after the exposure to the flame. Although the whisker is beneficial in making the char harder, it is not efficient in improving the ablation resistance due to the agglomerations of the whisker when the content is more than 6 phr. It is worth noting that the char layer of SiCw/MMT-filled samples was relatively not easy to detach from the ablated material when compared to the control and MMT-filled samples. Furthermore, the char of SiCw/MMT-filled samples seemed to be compact and stable, especially in the case of the SR16 composite, while the chars of the control and MMT-filled samples are quite porous and fragile. A compact and stable char layer is more effective as a heat shield to protect the inner part of the materials. On the basis of this analysis, it was concluded that the optimum content of SiCw in the composites was 6 phr as the second filler. The following part will focus on the ablation behavior of the SiCw/MMT-filled composite (SR16) compared with the control (SR00) and MMT-filled sample (SR10). 

**Figure 7:**
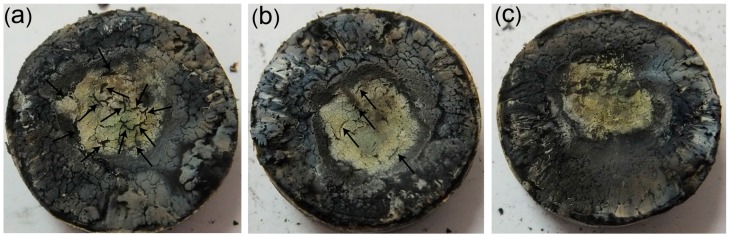
Photographs of ablated SR composites: (**a**) SR00; (**b**) SR10 and (**c**) SR16.

The digital photos of the ablated samples are shown in Figure 7. Many macro cracks (see the arrows) can be seen in the control sample and a very broad ablated center formed on the surface. Oxygen can spread through the penetrating cracks into the interior, which accelerated the oxidation of the polymers. The macro cracks of the ablated surface decreased to some extent in the case of the MMT-filled composite (SR10). After the addition of 6 phr SiCw, the macro cracks largely disappeared on the surface of the ablated composite. The oxyacetylene flame not only provided a high temperature heat but also reproduced a shear stress caused by combustion gases. In this condition, the decomposition of organic materials took place very quickly, and resulted in different thermal stress of the char; meanwhile, the char could be peeled off by the shear stress. These combined actions might appear as macro cracks in the ablated surface. Natali et al. [43] studied the ablated surface of EPDM-based materials, and considered that the char structure was related to the macro cracks and ablation efficiency. These observations indicated that the incorporation of SiCw/MMT was beneficial in decreasing the macro cracks of the ablated surface, in other words, a compact and stable char was obtained. 

**Figure 8:**
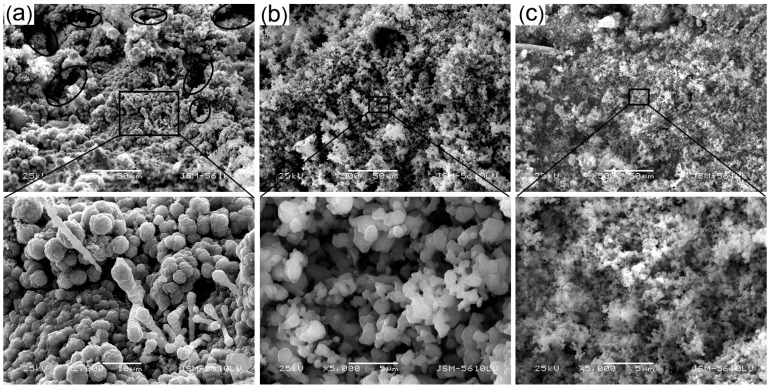
SEM micrographs for char surfaces of SR composites after oxyacetylene torch test: (**a**) SR00; (**b**) SR10 and; (**c**) SR16.

The morphologies of the char surface of the samples after oxyacetylene torch test are displayed in Figure 8. In the experimental test, the temperature of the sample surface was about 2200 °C, so the decomposition of the matrix occurred very quickly. In this oxidizing environment, the fiber also reacted with the oxygen in the combustion gases, so the integrating effect of fiber in the char layer was weakened. As seen in Figure 8a, the control sample showed an uneven morphology with some disordered cavities (see the circles). Although the melting of the silica can absorb heat, it is incapable of protecting the internal material. The char will peel off of the ablated center and the inner layer is then exposed to the oxyacetylene flame, which leads to further decomposition and oxidation, causing limited ablation resistance. Under high magnification, the carbonized fiber is visible. By contrast, there is a covering layer in the ablated surfaces after the introduction of the anti-ablation particles and some remaining fibers are also evident under this layer. The melting temperature of the MMT is about 1200 °C, and the viscous film of the molten MMT acts as a high-temperature binder for the silica and other components, further protecting the char layer and the interior materials against the oxyacetylene flame [44]. The covering layer of char can serve as a heat shield to decelerate the diffusion of heat and oxygen into inner layers and the remaining fiber can also maintain the char strength. Under high magnification, it can clearly be seen that the particles adhered and bonded to each other. However, the covering layer of the SR10 composite was porous due to the diffusion of the pyrolysis gases of the organic materials. The ablated surface of the SR16 composite showed no obvious porous morphology, and the covering layer seemed to be more compact than the SR10 composite. This covering layer was composed by many nanoparticles and submicron particles sintered as a dense and compact mass, as shown by high magnification. 

**Figure 9:**
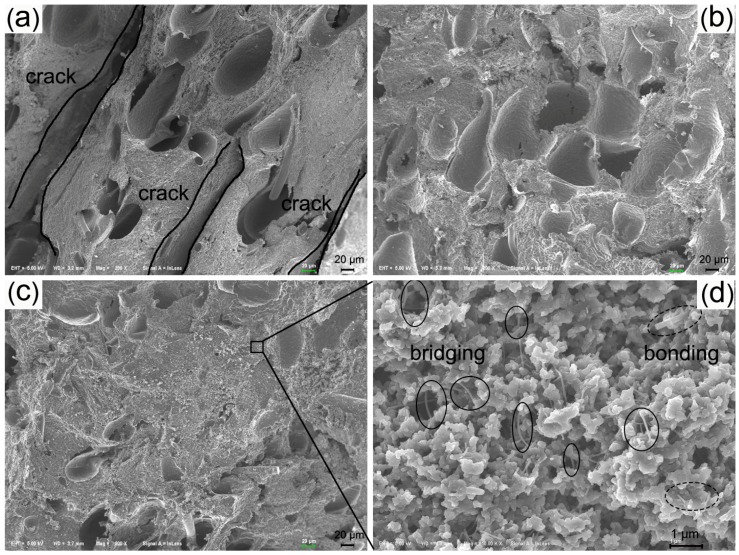
Cross section morphologies of char layer of SR composites after oxyacetylene torch test: (**a**) SR00; (**b**) SR10; (**c**) SR16 and; (**d**) high magnification of the selected region.

The cross section morphologies of the char layer of SR composites after an oxyacetylene torch test are shown in Figure 9. For the control sample, obvious cracks and pores can be observed, which resulted from the accumulation of pyrolysis gases in the inner part of the material. If the char was not sufficiently strong, the different thermal stress in the longitudinal direction would induce the char to delaminate to form cracks. The char of the SR10 composite was continuous with no evident cracks, while the pore size was larger than that of the control sample. The addition of two-dimensional MMT and the formation of a viscous binder were considered to provide a barrier effect, slowing the diffusion of pyrolysis gases, which could increase the accumulation of gas in the char layer [14,44]. This is also evidence for the low gas permeability, which would effectively retard the oxygen diffusion into the inner part of the material. The char layer of the SR16 composite was far denser and harder than the control and SR10 samples, although the pores were still in evidence. SiC was applied as an oxidation inhibitor to consume the oxygen and improve the ablation properties at high temperature. When the temperature was above 1700 °C, Equations (3) and (4) played the leading roles. The produced gaseous phases such as SiO and CO were disadvantageous to the prevention of further oxidation. When the temperature was below 1700 °C, the oxidation of SiCw generated a silica coating (Equation (5)), which was beneficial to ablation performance [45,46]. The melting of the MMT can absorb the heat and decrease the temperature of the inner layers; moreover, the viscous binder that formed had low gas permeability. These conditions were favorable to the Equation (5), leading to extended retention of the whisker shape.

(3) $$\text{SiCw}\left(s\right)+{\text{O}}_{2}\;=\text{SiO}\left(g\right)+\text{CO}\left(g\right)$$

(4) $$\text{SiCw}\left(s\right)+{2\text{SiO}}_{2}\left(l\right)=3\text{SiO}\left(g\right)+\text{CO}\left(g\right)$$

(5) $$2\text{SiCw}\left(s\right)+{3\text{O}}_{2}\left(g\right)={2\text{SiO}}_{2}\left(l\right)+2\text{CO}\left(g\right)$$

The oxidized SiCw with a specific length-to-diameter ratio had a degree of compatibility and wettability that was due to the resulting silica coating, which provided a good adhesion to the char, causing it to act as a micro skeleton to maintain the strength of the char layer. As seen in Figure 9d, the dimensions of most of the whiskers were below 100 nm, which reflected the oxidation of the whiskers. The bridging (the circles in Figure 9d) and bonding (the dot circles in Figure 9d) effects of the whiskers contributed to significant reinforcement of the char layer [33,34,35]. 

**Figure 10:**
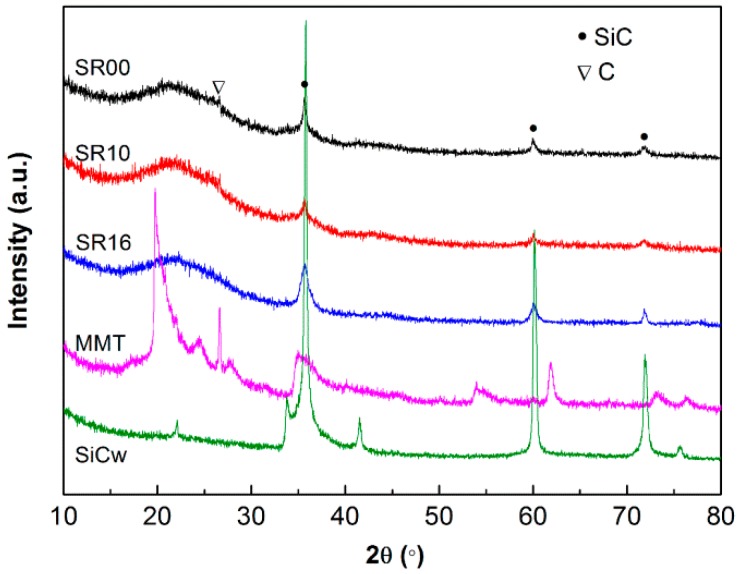
XRD patterns for MMT, SiCw and different char layer powders of SR composites after an oxyacetylene torch test.

**Figure 11:**
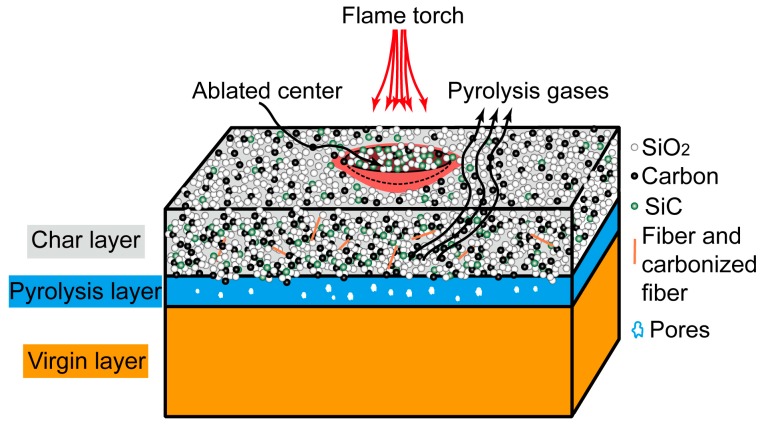
A schematic representation of the ablation process of basic SR composite.

Figure 10 shows the XRD patterns of the MMT, SiCw and char powders. The appearance of a large hump centered at 2θ = 22° is the typical amorphous diffraction peak of SiO_2_ [47]. A small hump appeared at 26.8, which corresponds to the diffraction peak of carbon. The diffraction peaks at 35.6°, 62° and 73° are associated to SiC crystal structure, indicating the in situ formation of SiC particles. This SiC may be formed by carbothermal reduction of SiO_2_ and C according to reactions (6)–(8) [15,48,49], and a schematic representation of the ablation process of basic SR composite is shown in Figure 11.

(6) $$\text{MVS}+\text{MPVS}\to {\text{SiO}}_{2}\left(s\right)+\text{C}\left(s\right)+\text{VOC}\left(g\right)$$

(7) phenolic →C(s)+VOC(g)

(8) $${\text{SiO}}_{2}\;\left(s\right)+3\text{C}\left(s\right)=\text{SiC}\left(s\right)+2\text{CO}\left(g\right)$$

The in situ-formed SiC was a thermally resistant ceramic, which was beneficial to the ablation performance of the SR composites. As seen in Figure 10, the MMT characteristic peaks did not appear, suggesting that any retained MMT in the char layer became amorphous. The layered structure of the MMT was destroyed and underwent a phase change, including melting and vaporization, which absorbed a large amount of the heat during the ablation [50]. For the char of SR16 composite, the diffraction peak associated with SiC was sharper than those of the composites SR00 and SR10 due to the remaining thermally resistant SiCw filler. 

## 4. Conclusions

In this study, the effects of MMT and SiCw/MMT on the mechanical, thermal and ablation properties of silicone rubber composites were investigated. The incorporation of MMT slightly increased the tensile strength and elongation of the resulting composite. The linear and mass ablation rates decreased by 22.5% and 18.2%, respectively, compared to the control sample. When subjected to a high temperature flame, the MMT in the composite experienced a phase change, including melting and vaporization, which absorbed a large amount of the heat and formed a covering layer by bonding with silica and other material components, which protected the inner layers from being ablated by the oxyacetylene flame. When SiCw was added to the MMT composite as the second filler, the tensile strength of the composite exhibited a significant increase, while the elongation at break decreased. The SiCw was wettable by the char due to the formed silica coating and may have served as a micro skeleton that maintained the strength of the char. The linear and mass rates of the SR16 composite were 0.049 mm/s and 0.031 g/s, respectively, corresponding to decreases of 59.2% and 43.6% over the control sample. Moreover, the density of all the fabricated composites was less than 1.20 g·cm^−3^.

## Figures and Tables

**Table 1:** Formula of the silicone rubber (SR) composites. MVS: Methyl vinyl silicone rubber; MPVS: methyl phenyl vinyl silicone rubber; DBPMH: 2,5-bis(tert-butyl peroxy)-2,5-dimethyl hexane; MMT: montmorillonite.

Sample	Content (phr) ^a^						
	MVS	MPVS	SiO_2_	Kynol	DBPMH	MMT	SiCw
SR00 (control)	40	60	30	10	1.5	0	0
SR10	40	60	30	10	1.5	10	0
SR12	40	60	30	10	1.5	10	2
SR14	40	60	30	10	1.5	10	4
SR16	40	60	30	10	1.5	10	6
SR18	40	60	30	10	1.5	10	8

^a^ Parts per hundred parts of SR (phr).

**Table 2:** Mechanical parameters and density of SR composites.

Sample	Tensile Strength (MPa)	Elongation at Break (%)	Shore A	Density (g·cm^−3^)
SR00	4.23 ± 0.26	603 ± 6	65.1	1.081
SR10	4.72 ± 0.35	611 ± 5	67.5	1.114
SR12	5.08 ± 0.31	575 ± 9	72.3	1.121
SR14	5.20 ± 0.23	562 ± 11	73.8	1.136
SR16	5.61 ± 0.31	520 ± 7	75.2	1.143
SR18	5.28 ± 0.24	502 ± 13	75.5	1.159

**Table 3:** Characteristic TGA temperature data of SR composites measured in air atmosphere.

Sample	T_0.1_ (°C)	T_max_ (°C)	R_800_ (%)
SR00	418.5	515.0	29.58
SR10	433.1	523.1	29.59
SR12	449.8	524.8	33.69
SR14	445.6	523.1	33.69
SR16	442.6	521.1	34.11
SR18	436.1	518.6	34.68
